# Emodin from *Aloe* Inhibits *Porcine Reproductive and Respiratory Syndrome Virus* via Toll-Like Receptor 3 Activation

**DOI:** 10.3390/v13071243

**Published:** 2021-06-26

**Authors:** Zhichao Xu, Meiyan Huang, Yongbo Xia, Peng Peng, Yun Zhang, Shumei Zheng, Xiaowei Wang, Chunyi Xue, Yongchang Cao

**Affiliations:** 1 State Key Laboratory of Biocontrol, School of Life Science, Sun Yat-sen University, Guangzhou 510006, China; xuzhich5@mail.sysu.edu.cn (Z.X.); huangmy53@mail2.sysu.edu.cn (M.H.); xiayb5@mail2.sysu.edu.cn (Y.X.); pengp9@mail2.sysu.edu.cn (P.P.); zhangyun6@mail.sysu.edu.cn (Y.Z.); zhengshm9@mail2.sysu.edu.cn (S.Z.); wangxw67@mail2.sysu.edu.cn (X.W.); xuechy@mail.sysu.edu.cn (C.X.); 2 School of Life Science, Sun Yat-sen University, Higher Education Mega Center, Guangzhou 510006, China

**Keywords:** *Porcine reproductive and respiratory syndrome virus* (PRRSV), Emodin, antiviral activity, Toll-like receptor 3 (TLR3), type I interferon

## Abstract

*Porcine reproductive and respiratory syndrome virus* (PRRSV) causes severe reproductive failure in sows and respiratory diseases in growing and finishing pigs and results in great economic losses to the swine industry. Although vaccines are available, PRRSV remains a major threat to the pig farms. Thus, there is an urgent need to develop antiviral drugs to compensate for vaccines. In this study, we report that *Aloe* extract (Ae) can strongly inhibit PRRSV in Marc-145 cells and porcine alveolar macrophages lines (iPAMs) in vitro. Furthermore, we identified a novel anti-PRRSV molecule, Emodin, from Ae by high-performance liquid chromatography (HPLC). Emodin exerted its inhibitory effect through targeting the whole stages of PRRSV infectious cycle. Moreover, we also found that Emodin can inactivate PRRSV particles directly. Notably, we confirmed that Emodin was able to significantly induce Toll-like receptor 3 (TLR3) (*p* < 0.01), IFN-α (*p* < 0.05) and IFN-β expression in iPAMs, indicating that induction of antiviral agents via TLR3 activation by Emodin might contribute to its anti-PRRSV effect. These findings imply that the Emodin from *Aloe* could hamper the proliferation of PRRSV in vitro and might constitute a new approach for treating PRRSV infection.

## 1. Introduction

Porcine reproductive and respiratory syndrome (PRRS) caused by *Porcine reproductive and respiratory syndrome virus* (PRRSV), is one of the most important viral diseases in the swine industry. PRRSV belongs to the genus *Arterivirus* of the family *Arteriviridae* [1], which also comprises *Lactate dehydrogenaseelevating virus* (LDV), *Equine arteritis virus* (EAV) and *Simian hemorrhagic fever virus* (SHFV) [2]. PRRSV is an enveloped, single-stranded, positive-sense RNA virus, whose genome is approximately 15.4 kb in size, and arranged in the order of: 5′ UTR-ORF1a-ORF1b-ORF2a-ORF2b-ORF3-ORF4-ORF5-ORF6-ORF7-3′ UTR, encoding 14 non-structural proteins and 8 structural proteins [3,4,5]. PRRSV can be classified into the European genotype (type 1) and the North American genotype (type 2) [6]. PRRSV can replicate in the macrophages, inducing prolonged viremia and persistent infection [7]. Marc-145, a stable African green monkey embryonic kidney cell line, is also used in PRRSV culture in vitro [8]. Since PRRS was first reported in United States in 1987, this disease has spread soon to other regions of the world [9]. Of note, there have been devastating outbreaks of highly pathogenic PRRSV in China in 2006, which is characterized by high fever, high morbidity and high death rates in pigs [10], leading to substantial economic losses to the swine industry.

To combat the virus, substantial efforts have been made to prevent and control PRRSV infection in the pig farms. For PRRSV infection prevention, vaccination is the present dominant management strategy. Currently, several vaccines including inactivated vaccine [11], modified-live vaccine [12], recombinant vector vaccine [13], DNA vaccine [14] and subunit vaccine [15] are widely used to prevent PRRSV infection. Unfortunately, it still cannot stop PRRS outbreaks, due to certain drawbacks of those vaccines with respect to safety and efficacy [9]. In addition, CD163-edited pigs have been shown to be able to resist PRRSV infection [16], but this is still in the research stage. Antiviral agents can be used in either a therapeutic or a prophylactic mode to complement vaccines. Previous studies have discovered a few natural compounds and compositions such as *Cryptoporus volvatus* Extract [2], Glycyrrhizin [17], Flavaspidic acid AB [6] and Tea Polyphenols [18] that have anti-PRRSV activities. However, there is no distinct commercial antiviral drug available for treating pigs after PRRSV infection.

It has been reported that natural products can be used as a source of new generations of antiviral agents [19]. *Aloe vera* has significant inhibitory effect on a wide range of viruses, such as *Herpes simplex virus type 1*, *Influenza virus* and *Pigeon paramyxovirus type 1* [20,21,22]. Antiviral effects have been described not only for whole extracts of *Aloe vera*, but also for their isolated compounds. Emodin (1, 3, 8-trihydroxy-6-methylanthraquinone) is a derived anthraquinone compound isolated from roots and barks of pharmaceutical plants, including *Rheum palmatum* and *Aloe vera* [23]. It has been reported that Emodin has a significant inhibitory effect on *Cyprinid herpesvirus 3* [24], *Coxsackieviruses* [25,26,27], *Zika virus* [28], *Enterovirus 71* [29], *Epstein-Barr virus* [30], *HCoV-OC43* [31], *Herpes simplex virus* [32,33], *Hepatitis B virus* [34] and *SARS-CoV* [35]. The antiviral activity of Emodin achieved by blocking the virus-receptor interaction [35], inhibiting viral protein translation [26], viral maturation [29], and viral release [31].

Although *Aloe* and its component Emodin could serve as an antiviral agent against many viruses, detailed information about the antiviral efficacy against PRRSV remains unclear. In this study, we first investigated the antiviral activity of Ae against PRRSV infection in vitro. Furthermore, we examined the potential of Emodin from *Aloe* to inhibit PRRSV replication in vitro and determined the stages in the PRRSV life cycle that could be blocked by Emodin, and then analyzed the possible anti-PRRSV mechanism of Emodin. Our data showed that Emodin from Ae was able to effectively inhibit PRRSV infection in vitro, and TLR3 activation by Emodin might be associated with its anti-PRRSV effect, indicating that a therapeutic potential of Emodin against PRRSV.

## 2. Materials and Methods

### 2.1. Cells, Virus and Virus Preparation

Marc-145 cells were obtained from Wen’s Foodstuffs Group Co., Ltd. (Guangdong, China) and porcine alveolar macrophages lines (iPAMs) were kindly provided by Professor Shaobo Xiao (Huazhong Agricultural University, Wuhan, China). Marc-145 cells and iPAMs were cultured in Dulbecco’s modified eagle medium (DMEM) (Hyclone, Logan, UT, USA), RPMI medium modified (Hyclone), respectively. Both media were supplemented with 100 U/mL penicillin, 100 U/mL streptomycin, and 10% fetal bovine serum (FBS) (BOVOGEN, East Keilor, Australia). The maintenance medium was DMEM or RPMI medium supplemented with 2% FBS.

Virus propagation was performed as previously described with some modifications [8]. Briefly, one milliliter of highly pathogenic PRRSV Li11 strain together with 20 mL fresh maintenance medium was added to T175 flask after Marc 145 cells reached 100% confluence. The virus-inoculated cells were cultured continuously at 37 °C in 5% CO_2_ for around 2–3 days. When >80% cytopathic effect (CPE) was evidently observed, the flasks were twice frozen at −80 °C and thawed to harvest the cells and supernatants. Virus titers were determined using the Reed-Muench method [36] and expressed as tissue culture infective dose 50% (TCID_50_). Plaque forming unit (PFU) was determined according to “PFU = 0.7 × TCID_50_” as described previously [37], and the multiplicity of infection (MOI) was calculated based on PFU.

### 2.2. High-Performance Liquid Chromatography (HPLC) Analysis of Ae

The dried powder of Ae was purchased from Bioforte Biotechnology Co., Ltd. (Shenzhen, China). One hundred milligrams (mg) was dissolved with 1 milliliter (mL) DMEM and filtered with 0.22 μm filters and then stored at −4 °C until use. Reference standards: Aloin, Quercetin and Emodin isolated from *Aloe* were purchased from the National Institutes for Food and Drug Control (Beijing, China). Ae was identified using a Waters e2695 HPLC system (Waters Corporation, Milford, CT, USA) equipped with a diode array detector as previously described with some modifications [38]. Briefly, chromatographic separation was achieved on an Agilent ZORBAX SBC18 HPLC column (4.6 mm × 250 mm, 5 μm) at 35 °C. The mobile phase composition was acetonitrile-water for Aloin (PubChem CID: 313325), methanol-0.4% orthophosphoric acid solution for Quercetin (PubChem CID: 5280343), methanol-0.1% orthophosphoric acid solution for Emodin (PubChem CID: 3220). Detection wavelengths were 355 nm for Aloin, 360 nm for Quercetin, and 254 nm for Emodin.

### 2.3. CCK-8 Assay

The Cell Counting Kit-8 (CCK-8) (Yeasen Biotech, Shanghai, China) was used to examine the cytotoxicity of drugs on Marc-145 and iPAM cells. Briefly, Aloin, Emodin and Quercetin were dissolved with dimethylsulfoxide (DMSO) at concentrations of 400 μg/mL, 100 μg/mL and 400 μg/mL, respectively. 100% confluent cells in 96-well plates were incubated with the control normal maintenance medium or the maintenance medium containing 0.1% DMSO or various concentrations of drugs (Ae, Aloin, Emodin and Quercetin) for 24 h and 48 h. Then, 100 μL maintenance medium containing 10% CCK-8 reagent was added to each well, and the cells were incubated at 37 °C for another 1 h before the CCK-8 signal was measured at an absorbance of OD_450 nm_. The relative viability of the cells was analyzed according to “cell survival rate (%) = [OD (sample) − OD (blank)/OD (control) − OD (blank)] × 100%”.

### 2.4. PRRSV Infection and Drugs Treatment In Vitro

Marc-145 or iPAM cells were seeded on 12-well plates and cultured for 90% confluence before inoculation with various concentrations of drugs (Ae, Aloin, Emodin and Quercetin), or the control normal maintenance medium or the maintenance medium containing 0.1% DMSO for 1 h, followed by infection with PRRSV at a MOI of 0.1 for 1 h, and then fresh maintenance medium containing different concentrations of drugs was added after the viral inoculums were removed. Twenty-four hours later, indirect immunofluorescence assay (IFA) was performed as described below. Cells were collected and cell lysates were prepared at indicated time points (12 h, 24 h or 48 h). Western Blot, as described below, and TCID_50_ analysis, as described above, were performed to examine the expression of N and GAPDH at a protein level and the viral titers, respectively.

### 2.5. Indirect Immunofluorescence Assay (IFA)

IFA was performed to observe PRRSV-infected cells as described previously with some modifications [39]. Briefly, cells were fixed with 4% paraformaldehyde for 15 min at 4 °C and then permeabilized with 0.2% Triton X-100 for 15 min at room temperature. After blocking with 1% bovine serum albumin (BSA), PRRSV N protein in cells was detected with mouse monoclonal antibody against PRRSV N protein (Beijing Jinnuo Baitai Biotechnology Co., Ltd., Beijing, China) (1:1000) and Cy3-labeled goat anti-mouse secondary antibody (KPL, Maryland, USA) (1:500). After three washes with 1× phosphate buffer saline (PBS), nuclei were counter-stained with 4,6-diamidino-2-phenylindole (DAPI). The immunofluorescence was observed using a fluorescence microscope (NIKON Eclipse 80i, Tokyo, Japan).

### 2.6. Western Blot Analysis

The drug-treated PRRSV-infected or control whole-cells were lysed in RIPA lysis buffer (Beyotime, Shanghai, China) containing 1% protease inhibitors (Yataihengxin, Beijing, China). The supernatant was collected after centrifugation and boiled with 6× SDS loading buffer for 10 min. Equivalent volume of protein samples was separated by 15% sodium dodecyl sulfate-polyacrylamide gels (SDS-PAGE) electrophoresis, and electroblotted onto polyvinylidene fluoride (PVDF) membranes (Millipore, New Jersey, USA). Membranes were blocked with 5% skim milk, then incubated with mouse monoclonal antibody against PRRSV N protein (Beijing Jinnuo Baitai Biotechnology Co., Ltd., Beijing, China) (1:500) or mouse monoclonal antibody against GAPDH (Proteintech Group, Inc., Chicago, IL, USA) (1:5000) and HRP-conjugated goat anti-mouse IgG (Proteintech Group, Inc., Chicago, IL, USA) (1:8000). The blots were detected using the enhanced chemiluminescent (ECL) reagent (Fdbio, Hangzhou, China).

### 2.7. Time Course Analysis of Emodin Anti-PRRSV

Confluent monolayers of iPAMs in 12-well plates were inoculated with PRRSV Li11 (MOI = 0.1) at 4 °C for 1 h to synchronize infection, and then 1 mL of maintenance medium was added to each well after the viral inoculums were removed. Cells were further incubated at 37 °C. An Emodin solution (6.25 μg/mL) was added to the wells or to the virus samples at various time points. After 24 h, real-time PCR, as described below, and Western Blot, as described above, were performed to examine the mRNA and protein levels of PRRSV N in the cells. A TCID_50_ assay was performed as described above to determine the viral titers in the cell lysates.

### 2.8. RNA Extraction and Real-Time PCR Analysis

Total viral RNA was extracted from iPAMs after PRRSV infection using a RNeasy kit (Magen, Guangzhou, China) and 450 ng RNA was used for cDNA synthesis using RT-PCR kit (TaKaRa, Dalian, China). Real-time PCR assay was performed using the specific primers for PRRSV nucleocapsid (*N*) gene (sense: 5′-TAAGATCATCGCCCAACAAA-3′; antisense: 5′-TCGGCAAACTAAACTCCACA-3′), and glyceraldehyde-3-phosphate dehydrogenase (*GAPDH*) (sense: 5′-CCTTCCGTGTCCCTACTGCCAAC-3′; antisense: 5′-GACGCCTGCTTCACCACCTTCT-3′) [40] by a Light Cycler 480 (Roche, Basel, Switzerland). Each PCR reaction was carried out in a 10-μL volume containing 1 μL of cDNA, 5 μL 2× PerfectStartTM Green pPCR SuperMix (TransGen Biotech, Beijing, China), and a 0.4 μM of each gene-specific primer. The thermal cycling parameters were as follows: 95 °C for 5 min; 40 cycles of 95 °C for 10 s, 58 °C for 10 s, and 72 °C for 30 s; and 1 cycle of 95 °C for 5 s, 65 °C for 1 min, and 95 °C for 15 s. The final step was to obtain a melt curve for the PCR products to determine the specificity of the amplification. All samples were tested in triplicate on the same plate, and the amplified products were calculated using the comparative threshold cycle (Ct) method. The mRNA expression levels of *N* gene were normalized to the expression of the *GAPDH* gene.

To analyze the signal transduction pathways activated by Emodin after PRRSV infection, total RNA was extracted from iPAMs using a RNeasy kit (Aidlab, Beijing, China) and 450 ng RNA was converted to cDNA by using RT-PCR kit (TaKaRa, Dalian, China). Real-time PCR assay was performed using the specific primers for porcine *TLR3* (sense: 5′-TAACAACCTTCCAGGCATA-3’; antisense: 5′-AAGAGGAGAATCAGCGAGTG-3’), *IFN-α* (sense: 5′-TCTCATGCACCAGAGCCA-3′; antisense: 5′-CCTGGACCACAGAAGGGA-3′), *IFN-β* (sense: 5′-AGTGCATCCTCCAAATCGCT-3′; antisense: 5′-GCTCATGGAAAGAGC TGTGGT-3′), and *GAPDH* (sense: 5′-CCTTCCGTGTCCCTAC TGCCAAC-3′; antisense: 5′-GACGCCTGCTTCACCACCTTCT-3′) [40,41] by a Light Cycler 480 (Roche). The PCR was performed in a 10-μL volume containing 1 μL of cDNA, 5 μL 2× PerfectStartTM Green pPCR SuperMix (TransGen Biotech), and a 0.4 μM of each gene-specific primer. The amplification conditions and the melt curve were as described above. The mRNA expression levels of *TLR3*, *IFN-α*, *IFN-β* were normalized to the endogenous level of the reference gene *GAPDH* and expressed as fold increase or decrease relative to the control samples.

### 2.9. Direct Virion Inactivation Activity of Emodin Analysis

The efficacy of Emodin to inactivate PRRSV directly was determined as previously described with some modifications [39]. Briefly, PRRSV strain Li11 of 3.5 × 10^6^ PFU was mixed with Emodin at a concentration of 6.25 μg/mL for 3 h at 37 °C. Following the treatment, TCID_50_ assay was performed as described above to determine the viral infectivity of the samples, and RT-PCR was performed using the specific primers to examine the viral genome as described previously [42]. 

### 2.10. Activation of Signal Transduction Pathways by Emodin after PRRSV Infection

iPAMs (1 × 10^5^) were seeded on a 6-well plate and cultured for 12 h before treatment with Emodin (6.25 μg/mL) or 0.1% DMSO for 1 h, and then infected with PRRSV at a MOI of 0.1. One hour after infection, the viral inoculums were removed and fresh maintenance medium containing Emodin (6.25 μg/mL) or 0.1% DMSO were added again. Only 0.1% DMSO or Emodin were added as controls. Twelve or twenty-four hours later, total RNA was extracted and used for cDNA synthesis. Real-time PCR was performed to examine the expression of pig TLR3, IFN-α, IFN-β and GAPDH at mRNA level as described above.

### 2.11. Statistical Analysis

Statistical analysis was performed using GraphPad Prism software 5.0 (GraphPad, San Diego, CA, USA), and differences among the experimental groups (cell viability, PFU, *N* mRNA, TLR3, IFN-α and IFN-β) were evaluated using ANOVA and Mann-Whitney, accordingly. *p*-values < 0.05 were considered statistically significant.

## 3. Results

### 3.1. Ae Inhibits PRRSV Infection In Vitro

**Figure 1:**
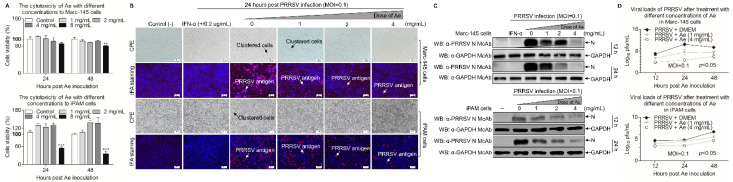
Ae inhibits PRRSV replication in vitro. (**A**) The cytotoxicity of Ae to Marc-145 and iPAM cells in vitro. Marc-145 or iPAM cells were cultured with various concentrations of Ae (2–8 mg/mL) or the control normal DMEM for 24 h and 48 h prior to the CCK-8 assay. (**B**) Marc-145 or iPAM cells were treated with various concentrations of Ae (1, 2 or 4 mg/mL) or the control normal DMEM for 1 h, followed infection with PRRSV at a MOI of 0.1. After 1 h, the cells were re-treated with Ae or DMEM as a control. At 24 h post-inoculation (hpi), an indirect immunofluorescence assay was performed. IFN-α was used as a positive control. CPE and PRRSV antigen were indicated by arrows. (**C**) Marc-145 or iPAM cells were treated as described above: at indicated time points (12 h and 24 h), cell lysates were prepared and examined with Western Blot using anti-PRRSV N monoclonal antibody and anti-GAPDH monoclonal antibody; or (**D**) the viral yield in the cell lysates were quantified by TCID_50_ analysis. Results are representative of three independent experiments (mean ± SD). *n* = 8 or 3. ** *p* < 0.01, *** *p* < 0.001.

To determine the potential cytotoxicity caused by Ae, we used CCK-8 assay to test the cytotoxicity of Ae at various concentrations in Marc-145 and iPAM cells. As shown in Figure 1A, the cells were incubated with 1–4 mg/mL Ae for 24 h and 48 h, the relative viability of cells was 100% compared with the controls. However, the cell viability decreased to 80% or 40% (*p* < 0.01 or *p* < 0.001) when the concentration of Ae was 8 mg/mL. We further explored the antiviral activity of Ae against PRRSV by IFA with PRRSV N protein-specific monoclonal antibody based on the maximum safe concentration of Ae in cells. As shown in Figure 1B, the PRRSV-specific immunofluorescence gradually decreased in infected cells as the concentrations of Ae increased, indicating that Ae inhibited PRRSV infection in a dose-dependent manner. To further determine the anti-PRRSV effect of Ae, Western Blot using specific antibodies against N and GAPDH was performed to examine the level of N protein in PRRSV-infected cells at 12 h and 24 h. As a result, the expression level of N protein gradually decreased in infected cells as the concentrations of Ae increased (Figure 1C). To evaluate the influence of Ae on the PRRSV infection activity, TCID_50_ assay was used to detect the changes of virus titers after Ae treatment at different concentrations. As shown in Figure 1D, Ae could decrease the viral titers, as compared to the control. Taken together, Ae possesses anti-PRRSV infection activity in vitro.

### 3.2. Emodin from Ae Inhibits PRRSV Infection In Vitro

**Figure 2:**
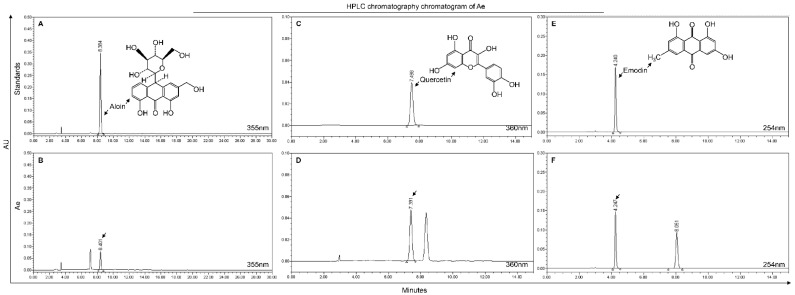
HPLC chromatography chromatogram of Ae. Detection at wave length 355 nm for Aloin from reference standard or Ae (**A**,**B**). Detection at wave length 360 nm for Quercetin from reference standard or Ae (**C**,**D**). Detection at wave length 254 nm for Emodin from reference standard or Ae (**E**,**F**).

To determine the anti-PRRSV component in Ae, we used HPLC to analyze the components in Ae. As shown in Figure 2, Aloin, Emodin and Quercetin were identified in the extracts of *Aloe vera*, and the retention times were 8.401, 4.247 and 7.391 min, respectively.

**Figure 3:**
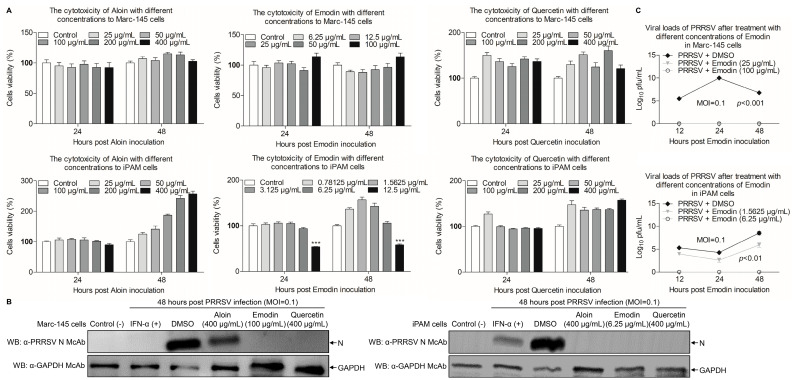
Emodin inhibits PRRSV replication in vitro. (**A**) The cytotoxicity of Aloin, Emodin and Quercetin to Marc-145 and iPAM cells in vitro. Marc-145 or iPAM cells were cultured with various concentrations of Aloin (25–400 μg/mL), Emodin (0.78125–100 μg/mL) and Quercetin (25–400 μg/mL) or the control 0.1% DMSO for 24 h and 48 h prior to the CCK-8 assay. (**B**) Marc-145 or iPAM cells were treated with various concentrations of Aloin (400 μg/mL), Emodin (6.25 or 100 μg/mL) or Quercetin (400 μg/mL) or the control 0.1% DMSO for 1 h, followed infection with PRRSV at a MOI of 0.1. After 1 h, the cells were re-treated with Aloin, Emodin or Quercetin and 0.1% DMSO as a control. At 48 hpi, cell lysates were prepared and examined with Western Blot using anti-PRRSV N monoclonal antibody and anti-GAPDH monoclonal antibody. (**C**) Marc-145 or iPAM cells were treated with Emodin as described above, at indicated time points (12 h, 24 h and 48 h), the viral titers in the cell lysates were detected by TCID_50_ analysis. Results are representative of three independent experiments (mean ± SD). *n* = 8 or 3. *** *p* < 0.001.

To investigate the potential cytotoxicity caused by these components of *Aloe*, CCK-8 assay was performed to test the cytotoxicity of Aloin, Emodin or Quercetin in Marc-145 and iPAM cells. As shown in Figure 3A, the relative viability of Marc-145 or iPAM cells was 100% after treatment with Aloin and Quercetin at the concentration of 400 μg/mL for 24 h and 48 h, as compared with the controls. Interestingly, the safe concentrations of Emodin in Marc-145 and iPAM cells were 100 μg/mL, 6.25 μg/mL, respectively, indicating that different components have different cytotoxicity in the same cells, and the same component can have different cytotoxicity in different cells. After determining the maximum safe concentrations of Aloin, Emodin and Quercetin in cells, we further used Western Blot to explore the influence of Aloin, Emodin or Quercetin on PRRSV. As shown in Figure 3B, the expression level of N protein decreased in infected cells receiving Aloin, Emodin or Quercetin treatment, as compared to the controls. However, the expression level of N protein after Emodin and Quercetin treatment decreased more significantly than Aloin treatment. These results suggest that these components of *Aloe* have anti-PRRSV effects, and Emodin and Quercetin have better anti-PRRSV effects than Aloin. Considering that the anti-PRRSV effect of Quercetin has been confirmed [43], we chose Emodin for the next step of anti-PRRSV study. To evaluate the influence of Emodin on the PRRSV infection activity, TCID_50_ assay was performed to detect the changes of virus titers after Emodin treatment. As shown in Figure 3C, the viral titers significantly decreased in the existence of Emodin (*p* < 0.01), as compared to the control. Taken together, Emodin from Ae has anti-PRRSV activity in vitro.

### 3.3. Emodin Acts at Whole Stages the Replication Cycle of PRRSV

**Figure 4:**
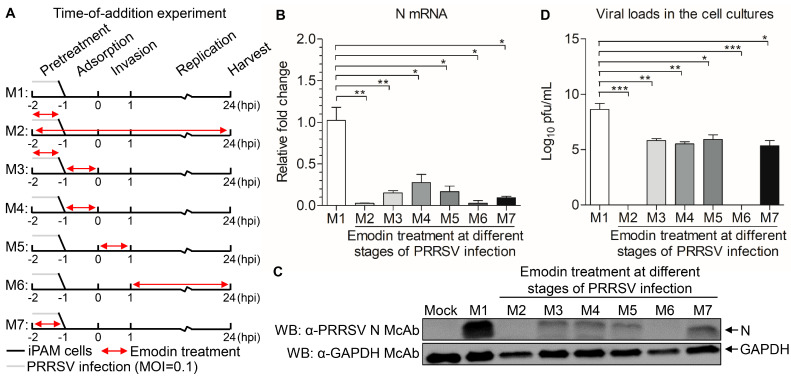
Effect of Emodin treatment on the replication stages of PRRSV. (**A**) iPAMs infected PRRSV were treated with Emodin (6.25 μg/mL) or 0.1% DMSO at different stages (M1-M7) of infection. Cells were harvested at 24 hpi, then the viral yields were titrated by (**B**) real-time PCR using specific primers, (**C**) Western Blot using anti-PRRSV N monoclonal antibody and anti-GAPDH monoclonal antibody and (**D**) TCID_50_ assay. The results are representative of three independent experiments (mean ± SD). *n* = 3. * *p* < 0.05, ** *p* < 0.01, *** *p* < 0.001.

To determine where the Emodin blocks the PRRSV infection, a time course analysis was established to determine the stage in which Emodin exhibits anti-PRRSV activity. As shown in Figure 4A, M1 stands for no Emodin treatment as control. M2 stands for the virus and cells treated with Emodin throughout the infection process. M3 stands for PRRSV pretreated with Emodin, with Emodin added in the virus adsorption process. M4 indicates that Emodin was added during the virus adsorption process. M5 indicates that Emodin was added in the virus invasion process. M6 indicates that Emodin was added in the virus replication process. M7 indicates that the cells were pretreated with Emodin. Twenty-four hours after PRRSV infection, cells were harvested to measure the mRNA and protein expression levels of N, and the viral tiers. As shown in Figure 4B–D, the mRNA and protein expression levels of N and viral titers all significantly decreased in M2, M3, M4, M5, M6 and M7 treatment groups, as compared to the M1 control group, indicating that Emodin has an antiviral effect on all stages of the PRRSV infectious cycle.

### 3.4. Viricidal Effect of Emodin on PRRSV Viral Particles

**Figure 5:**
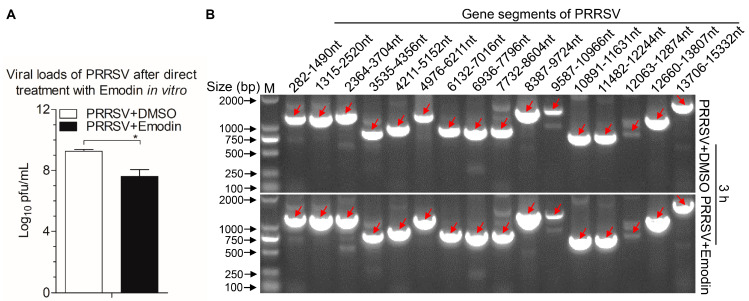
Emodin inactivated PRRSV directly but did not degrade the viral genome. PRRSV exposed to Emodin at indicated concentration (6.25 μg/mL) at 37 °C for 3 h, then virus yields were titrated by TCID_50_ in Marc-145 cells (**A**), the whole genome sequences of the PRRSV after Emodin or 0.1% DMSO treatment were amplified by RT-PCR (**B**). Data are representative of three independent experiments (mean ± SD). *n* = 3. * *p* < 0.05.

To investigate whether Emodin can directly inactivate PRRSV viral particles, live PRRSV were treated with Emodin or 0.1% DMSO at 37 °C for 3 h. Subsequently, the infectivity of the treated viruses was detected by TCID_50_ assay. As shown in Figure 5A, exposure of PRRSV to Emodin exhibited virucidal effect (*p* < 0.05), indicating Emodin can kill PRRSV directly. To verify whether Emodin destroys the viral genome, RT-PCR was performed, and the results revealed that Emodin at the indicated concentration of 6.25 μg/mL did not degrade the viral genome, as compared to the controls (Figure 5B).

### 3.5. Emodin Stimulation of TLR3 Activation Might Contribute to Its Anti-PRRSV Activity

**Figure 6:**
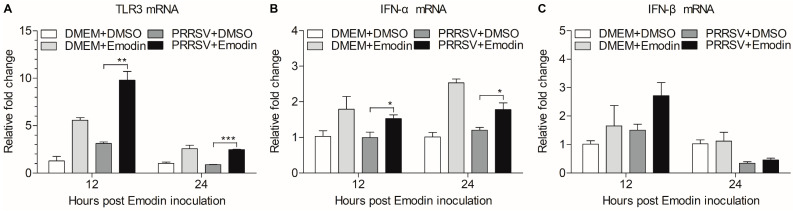
Emodin-induced activation of TLR3-mediated response after PRRSV infection. iPAMs were treated with Emodin (6.25 μg/mL) for 1 h, followed infection with PRRSV at a MOI of 0.1. After 1 h, the cells were re-treated with Emodin. Without Emodin treatment and without PRRSV infection, Emodin treatment only and PRRSV infection only as controls. At 12 hpi and 24 hpi, the mRNA expression levels of *TLR3* (**A**), *IFN-α* (**B**) and *IFN-β* (**C**) in iPAMs were examined by real-time PCR using specific primers. The mRNA expression levels of these molecules were normalized relative to the expression level of *GAPDH*. Data are represented as mean ± SD, *n* = 3. * *p* < 0.05, ** *p* < 0.01, *** *p* < 0.001.

TLR3 activation decreases PRRSV infection [44], which prompted us to examine the effect of Emodin on TLR3 after PRRSV infection. We found that Emodin could increase the mRNA expression of TLR3 (*p* < 0.01) in iPAMs after PRRSV infection at 12 hpi and 24 hpi, as compared to that of control (Figure 6A). It was reported that type I interferons (IFN-α/β) could be induced after TLR3 activation [45]. We further examined the mRNA expression of IFN-α and IFN-β in Emodin-treated iPAMs after PRRSV infection at 12 hpi and 24 hpi. As shown in Figure 6B,C, we found that Emodin was able to increase the mRNA of expression of IFN-α (*p* < 0.05) and IFN-β in iPAMs after PRRSV infection, indicating that Emodin stimulation of TLR3 activation-induced IFN-α might contribute to its anti-PRRSV activity.

## 4. Discussion

Since PRRSV was first reported to infect pigs, especially the emergence of highly pathogenic PRRSV in 2006 [10], it has caused significant problems in the swine industry. PRRSV infection causes reproductive failure, respiratory diseases, and high mortality in pigs [10]. Although vaccines are available for PRRSV, the delayed appearance and low titers of neutralizing antibodies, as well as a weak cell-mediated immune response after vaccination [46], result in large-scale infections despite vaccination. Antiviral drugs that are effective against PRRSV are urgently needed to combat these threats. In the present study, we report that Emodin from Ae inhibited PRRSV infection in vitro, which might help to control PRRS in the pig farms.

Plants are exploited extensively as candidates for new antiviral agents, due to the fact that they produce few side effects, and are abundant and cost-effective [47]. *Aloe vera* belongs to the *Liliaceae* family among the succulent plants, which possesses various properties such as being immunomodulatory, anti-inflammatory and antiviral in nature [48,49]. *Aloe vera* has a significant inhibitory effect on *Herpes simplex virus type 1*, *Influenza virus*, and *Pigeon paramyxovirus type 1* [20,21,22]. In addition, our previous study found that Ae can inhibit *Porcine epidemic diarrhea virus* (PEDV) in vitro and in vivo [39], which prompted us to determine whether Ae can resist PRRSV infection, another common virus in the pig farms. In our experiment, we found that Ae could inhibit PRRSV infection in Marc-145 and iPAM cells in vitro. However, whether Ae can also protect pigs against PRRSV in vivo needs further study.

The Ae used in this study, water extract from the body of *Aloe ferox*, is crude, containing many components, such as catechin hydrate and kaempferol, that have significant antiviral activity [50]. Although the antiviral effects of the extract could result from the mixture of active compounds rather than from a single chemical entity [2], it is necessary to determine the anti-PRRSV compounds in the Ae, because the extract also contains components that are harmful to the body [39]. In addition, identification of the anti-PRRSV components can help to develop new generation of antiviral agents. In this study, three components, Aloin, Quercetin and Emodin, were found in Ae by HPLC analysis (Figure 2), and these three components have all been previously reported to have antiviral effects [31,51,52]. We further found that these three components have anti-PRRSV effects, among which Quercetin and Emodin showed the best anti-PRRSV effects. Considering that the anti-PRRSV effect of Quercetin has been reported [43], we evaluated Emodin in detail. Emodin is an anthraquinone derivative, which has been reported to possess several biological properties, such as anti-inflammatory, anti-bacteria, anti-tumor, and immunosuppressive properties except anti-virus [53]. It is known that one problem of antiviral drugs is resistance, meaning that some viral particles survive from antiviral drugs, mutate and accumulate resistance to the antiviral drugs [54]. RNA viruses are more likely to gain resistance than DNA viruses, due to the higher mutational rates of RNA viruses than DNA viruses [55]. Interestingly, PRRSV was completely inhibited when the concentration of Emodin reached 6.25 μg/mL, indicating that Emodin can reduce the possibility of virus to develop resistance and may be used for a prolonged period. Although it is difficult to delineate how Emodin inhibits PRRSV infection, our results demonstrate that Emodin inhibits PRRSV infection at multiple steps of the virus life cycle including virus attachment and virus release (Figure 4). It has been reported that Emodin could significantly block the S protein-ACE2 interaction [35], indicating virus attachment between PRRSV and the receptors on the cell membrane might be blocked by Emodin. In addition, Emodin inhibits *HCoV-OC43* release by acting on SARS-associated coronavirus 3a protein [31], indicating that Emodin might also affect PRRSV release by acting on proteins associated with viral release, which needs more study in the future. We also found that Emodin could directly inhibit PRRSV infection activity in the absence of cells, but had no effect on viral genome, indicating that direct inactivation of the virus might be related to Emodin’s destruction of the viral envelope [56], but no on virus nucleic acid. 

TLR3 belongs to host pattern recognition receptors that can recognize double-stranded RNA (dsRNA) [40]. It has been reported that dsRNA stimulate expression of TLR3 contribute to suppress PRRSV infectivity in vitro and in vivo [44,57], indicating that TLR3 plays an important role in anti-PRRSV. In the present study, we found that the mRNA expression of TLR3 was induced by Emodin in iPAMs after PRRSV infection. On the contrary, Emodin could decrease the mRNA and protein levels of TLR3 and downstream molecules against *Coxsackievirus B3m* infection [25], indicating that Emodin has different antiviral mechanisms involved in fighting different virus infections. After recognition of viral nucleic acids, TLRs could recruit downstream kinases that phosphorylate downstream adaptor proteins to relay signals to activate transcription factors IFN regulatory factor 3 (IRF3) and IRF7, which are mainly involved in IFN gene induction [58]. IFN-α is often used as the positive control against PRRSV (Figure 3B). Our results demonstrate that Emodin could increase the mRNA expression of IFN-α (*p* < 0.05) and IFN-β in iPAMs after PRRSV infection, which might be the result of TLR3 activation, indicating that the anti-PRRSV effect of Emodin might be related to its stimulation of TLR3 activation leading to type I interferon expression. Collectively, all these findings confirm that Emodin from Ae can inhibit PRRSV replication and might serve as a good candidate to cure infection caused by PRRSV. However, there are still several important questions which need to be addressed. For example, what is the exact underlying mechanism of Emodin anti-PRRSV activity? Can Emodin resist PRRSV in vivo? In addition, does the combination of Aloin, Emodin and Quercetin have a better anti-PRRSV effect? Elucidation of these questions will help us to develop better strategy to control PRRSV infection.

## 5. Conclusion

Our data demonstrated that Emodin from *Aloe* suppresses PRRSV infection in vitro and exerts its antiviral effect by activating TLR3. Emodin might be developed into a novel antiviral agent and used to control PRRS in the pig farms.

## Data Availability

Obtained and analyzed data of this study are available from the corresponding author on request.
